# Proposal of a Tall Man Letter list for German-speaking countries

**DOI:** 10.1007/s00228-021-03091-3

**Published:** 2021-01-27

**Authors:** Johannes Heck, Adrian Groh, Dirk O. Stichtenoth, Olaf Krause

**Affiliations:** 1 Institute for Clinical Pharmacology, Hannover Medical School, Carl-Neuberg-Str. 1, 30625 Hannover, Germany; 2 Department for Psychiatry, Social Psychiatry and Psychotherapy, Hannover Medical School, Hannover, Germany; 3 Drug Commissioner, Hannover Medical School, Hannover, Germany; 4 Institute for General Medicine, Hannover Medical School, Hannover, Germany

Medication errors represent an avoidable threat to patient safety [1] and include—but are not limited to—administration of a drug to the wrong patient, choice of an incorrect route of administration, and administration of the false drug [2]. Administration of the false drug may result from the confusion of two similarly looking and/or sounding drug names, the so-called look-alike/sound-alike (LASA) medications [3]. We have previously reported on three cases of insidious LASA medication errors that occurred at our university hospital [4].

Since Hannover Medical School is obliged to a policy of transparent error management, the published cases [4] have been thoroughly investigated and potential countermeasures have been discussed to avoid similar scenarios in the future. Tall Man Lettering (TML) describes a concept of partial capitalisation of drug names that aims at better distinguishability, thereby reducing the risk of confusion of LASA medications [3, 5]. Internationally, several TML lists in English exist. In the USA, the Food and Drug Administration (FDA) has published a list with TML notations in 2001 as part of the agency’s name differentiation project [6]. The original FDA TML list has been amended by the Institute for Safe Medication Practices (ISMP) since 2008 [6]. ISMP Canada, in cooperation with the Canadian Association of Provincial Cancer Agencies, has published a similar TML list [7], yet with a focus on antineoplastic agents. An example for a TML list in a local language (i.e. Spanish) is the list by Otero López and colleagues [8].

In the aftermath of the disturbing cases of LASA medication errors that occurred at our institution [4], an interdisciplinary expert panel comprised of representatives from clinical pharmacology, psychiatry, and general medicine convened and decided to create a TML list in German. To this end, existing TML lists [6–8] were screened and appropriate agents were selected based on their availability and frequency of use in Germany. Medications predominantly prescribed by specialists, e.g. antineoplastic agents, were largely avoided in favour of more commonly used drugs such as antihypertensives, anti-infectives, and psychopharmaceuticals. For drugs used in emergency and intensive care medicine, TML recommendations in German already exist [9] and have been implemented at our university hospital. Of note, the drugs that had been confused in ref. [4] were incorporated into our list.

The final list which features TML notations in German for 44 LASA drug pairs/groups, comprising a total of 101 individual agents, was presented to, and approved by the Drug Commission of Hannover Medical School (Table 1 and Supplement 1). The list will be distributed to all newly employed physicians at our institution, accompanied by a brief description of the concept of TML. The primary goal of the list is the sensitisation of physicians to the problem of LASA medication errors. The list is a recommendation from physicians for physicians, its use is not mandatory. Physicians are encouraged to take advantage of TML in situations which bear a high risk of confusion of LASA drug names or if a problem with recurring confusion of certain LASA medications has been identified. Routine use of TML notations for every prescription, on the other hand, would be too time-consuming and is therefore not recommended. Even though the list has primarily been developed for handwritten prescriptions, it can also be incorporated into electronic prescription systems.

**Table 1:** Tall Man Letter list of Hannover Medical School, Hannover, Germany. The original German version is available as Supplement 1. Please note that drug names in German, as all nouns, start with a capital letter. TML, Tall Man Letter

Drug pair/drug group in German TML notation		Drug pair/drug group in German TML notation	
AMILorid	AmLODIPin	LevETIRAcetam — LevOCARNitin — LevoFLOXacin	
ARICept® (Donepezil)	AZILect® (Rasagilin)	LEVOmethadon	Methadon
AzaCITIDin	AzaTHIOprin	LORAtadin	LoVAStatin
BuPROPion	BuSPIRon	MELperon	MeroNEM® bzw. MeroPENEM
CefAZOLin — CefOTAXim — CefTAZIdim — CefTRIAXon — CefUROXim		MONurol® (Fosfomycin)	MOVIcol® (Macrogol 3350)
CITalopram	EScitalopram	NalTREXon	NaPROXen
CloBAZam — CloNIDin — CloPIDOgrel — CloZAPin		NexAVAR® (Sorafenib)	NexIUM® (Esomeprazol)
CLOTrimazol — CloMETHiazol — CoTRIMoxazol		PAZOPanib	PONATinib
ClomiFEN	ClomiPRAMIN	PenicillAMIN	Penicillin
CycloSERIN	CycloSPORIN	PENTobarbital	PHENobarbital
DACTINomycin	DAPTOmycin	PirACEtam	PirOXIcam
DiazePAM — DilaTREND® (Carvedilol) — DilTIAZem		PireTANid	PiriTRAMid
DimenhyDRINAT	DiphenhydrAMIN	PrednisoLON	PredniSON
DipiDOLOR® (Piritramid)	DipiPERON® (Pipamperon)	RifAMPicin	RifAXIMin
EbiXA® (Memantin)	EviSTA® (Raloxifen)	RisperDAL® bzw. RisperiDON — RoHYPnol® (Flunitrazepam) — ROPInirol	
EdoXABAN	ENDOxan® (Cyclophosphamid)	SandIMMUN® (Ciclosporin)	SandoSTATIN® (Octreotid)
EpiRUBIcin	EriBULin	SulfaDIAzin	SulfaSALAzin
FluCLOXAcillin	FluconAZOL	TerBINafin	TerFENAdin
FOSamax® (Alendronsäure)	TOPamax® (Topiramat)	TiaNEPtin	TiZANidin
HydrALAZIN — HydroCHLOROthiazid — HydroCORTison — HydroMORPHon — HydrOXYzin		TraMADol	TraZODon
IDArubicin	IdaruCIZUmab	ValACIclovir	ValGANciclovir
LaMICtal® bzw. LamoTRIgin — LamISIL® (Terbinafin) — LamiVUDin		ZyPREXA® (Olanzapin)	ZyrTEC® (Cetirizin)

The presented TML list (Table 1) may be used beyond our own institution in other countries where German is (one of) the official language(s), i.e. Austria, Belgium, Liechtenstein, Luxembourg, and Switzerland, as well as in countries with a language partly similar to German, e.g. the Netherlands and Scandinavian countries. Local adaptations regarding drug availability and frequency of use may be necessary. We strongly encourage colleagues in non-German-speaking countries to develop TML lists in the respective local language since TML is an acknowledged strategy to avoid LASA medication errors [3, 5].

## Supplementary Information

ESM 1 (PDF 63 kb)
