# Supplementary material for: Proposal of a Tall Man Letter list for German-speaking countries
Source: Eur J Clin Pharmacol. 2021 Jan 27;77(8):1247–9. doi: 10.1007/s00228-021-03091-3 (PMC8275545; doi:10.1007/s00228-021-03091-3)
Supplement: Supplementary file 1 — (PDF 63 kb) [file 228_2021_3091_MOESM1_ESM.pdf]

Dr. med. Johannes Heck[1], Dr. med. Adrian Groh[2],
PD Dr. med. Olaf Krause[3], Prof. Dr. med. Dirk. O. Stichtenoth[1]

[1]Institut für Klinische Pharmakologie
[2]Klinik für Psychiatrie, Sozialpsychiatrie und Psychotherapie
[3]Institut für Allgemeinmedizin

Medizinische Hochschule Hannover

Tall Man Letter Liste MHH

Tall Man Lettering (TML)[4] beschreibt ein Konzept der teilweisen Großschreibung von Medikamentennamen mit dem Ziel, eine Verwechslung ähnlich aussehender bzw. ähnlich klingender Medikamentennamen, sog. *look-alikes/sound-alikes* (LASA), zu vermeiden. Die nachfolgende Tabelle entstammt der klinischen Erfahrung der Autoren und soll auf das Problem der Verwechslung von LASA-Medikamenten aufmerksam machen. Wenngleich die routinemäßige Verwendung der TML-Schreibweise bei handschriftlicher Anordnung wenig praktikabel erscheint, so kann TML bei erkannten, wiederkehrenden Problemen hilfreich sein, um Medikamentenverwechslungen zu vermeiden. Aus der Tabelle wird deutlich, dass die Verschreibung von Handelspräparaten das Problem potenziert. Auf die Verschreibung von Handelspräparaten bzw. Markennamen sollte daher möglichst zugunsten von Freinamen verzichtet werden. In der Intensiv- und Notfallmedizin ist TML bereits seit längerer Zeit fest etabliert, entsprechende Medikamentenetiketten finden sich auf der Intranetseite der Zentralapotheke der MHH (https://intranet.mh-hannover.de/31448.html?&MP=135-8343).

Namenspaar bzw. -gruppe in TML-Schreibweise	
AMILorid	AmLODIPin
ARICept® (Donepezil)	AZILect® (Rasagilin)
AzaCITIDin	AzaTHIOprin
BuPROPion	BuSPIRon
CefAZOLin — CefOTAXim — CefTAZIdim — CefTRIAXon — CefUROXim	
CITalopram	EScitalopram
CloBAZam — CloNIDin — CloPIDOgrel — CloZAPin	
CLOTrimazol — CloMETHiazol — CoTRIMoxazol	
ClomiFEN	ClomiPRAMIN
CycloSERIN	CycloSPORIN
DACTINomycin	DAPTOmycin
DiazePAM — DilaTREND® (Carvedilol) — DilTIAZem	
DimenhyDRINAT	DiphenhydrAMIN
DipiDOLOR® (Piritramid)	DipiPERON® (Pipamperon)
EbiXA® (Memantin)	EviSTA® (Raloxifen)
EdoXABAN	ENDOxan® (Cyclophosphamid)
EpiRUBIcin	EriBULin
FluCLOXAcillin	FluconAZOL
FOSamax® (Alendronsäure)	TOPamax® (Topiramat)
HydrALAZIN — HydroCHLOROthiazid — HydroCORTison — HydroMORPHon — HydrOXYzin	
IDArubicin	IdaruCIZUmab
LaMICtal® bzw. LamoTRIgin — LamISIL® (Terbinafin) — LamiVUDin	

Namenspaar bzw. -gruppe in TML-Schreibweise	
LevETIRAcetam — LevOCARNitin — LevoFLOXacin	
LEVOmethadon	Methadon
LORAtadin	LoVAStatin
MELperon	MeroNEM® bzw. MeroPENEM
MONurol® (Fosfomycin)	MOVIcol® (Macrogol 3350)
NalTREXon	NaPROXen
NexAVAR® (Sorafenib)	NexIUM® (Esomeprazol)
PAZOPanib	PONATinib
PenicillAMIN	Penicillin
PENTobarbital	PHENobarbital
PirACEtam	PirOXIcam
PireTANid	PiriTRAMid
PrednisoLON	PredniSON
RifAMPicin	RifAXIMin
RisperDAL® bzw. RisperiDON — RoHYPnol® (Flunitrazepam) — ROPInirol	
SandIMMUN® (Ciclosporin)	SandoSTATIN® (Octreotid)
SulfaDIAzin	SulfaSALAzin
TerBINafin	TerFENAdin
TiaNEPtin	TiZANidin
TraMADol	TraZODon
ValACIclovir	ValGANciclovir
ZyPREXA® (Olanzapin)	ZyrTEC® (Cetirizin)

Korrespondenz:
Dr. med. Johannes Heck
Institut für Klinische Pharmakologie, OE 5350, Medizinische Hochschule Hannover
Tel.: +49 511 532-2725, Fax: +49 511 532-2750, E-Mail: heck.johannes@mh-hannover.de

[4]Bei TML handelt es sich um einen international etablierten Begriff. Die Bevorzugung eines bestimmten Geschlechts ist durch diesen Begriff nicht intendiert.

Version 1.0, November 2020
